# Volumetric MR-guided high-intensity focused ultrasound with direct skin cooling for the treatment of symptomatic uterine fibroids: proof of concept study

**DOI:** 10.1186/2050-5736-3-S1-O92

**Published:** 2015-06-30

**Authors:** Marlijne Ikink, Johanna van Breugel, Gerald Schubert, Robbert Nijenhuis, Lambertus W Bartels, Chrit Moonen, Maurice van den Bosch

**Affiliations:** 1 University Medical Center Utrecht, Utrecht, Netherlands; 2 Philips Healthcare, Vantaa, Finland

## Background/introduction

To prospectively assess the safety and technical feasibility of volumetric magnetic resonance-guided high-intensity focused ultrasound (MR-HIFU) ablation with direct skin cooling (DISC) during treatment of uterine fibroids.

## Methods

**Figure 1:**
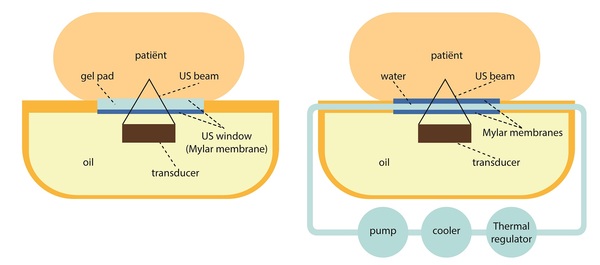


In this proof-of-concept study, eight patients were selected for clinical MR-HIFU ablation of uterine fibroids with use of an additional DISC device to maintain a constant temperature (T≈20°C) at the interface between the HIFU table-top and the patients’ skin (Figure 1). Technical feasibility was determined by verification of successful completion of MR-HIFU ablation. Contrast-enhanced T1-weighted MRI was used to measure the treatment effect (non-perfused volume (NPV) ratio). Safety was evaluated by recording of adverse events (AEs) and their relation to the investigational DISC device within 30 days’ follow-up.

## Results and conclusions

Results: All MR-HIFU treatments were successfully completed in an outpatient setting. The median NPV-ratio was 0.56 (IQR[0.27-0.72]). Immediately after treatment, two patients experienced coldness related discomfort which resolved the same day. No serious AEs were reported within 30-days’ follow-up. No skin burns, cold injuries or subcutaneous edema were observed in patients treated with the DISC device. Conclusion: This study showed that it is technically feasible and safe to complete a volumetric MR-HIFU ablation with DISC. This technique may further reduce the risk of thermal injury to the abdominal wall during MR-HIFU ablation of uterine fibroids.

